# Contrasting Regeneration Strategies in Climax and Long-Lived Pioneer Tree Species in a Subtropical Forest

**DOI:** 10.1371/journal.pone.0112385

**Published:** 2014-11-10

**Authors:** Haiyang Wang, Hui Feng, Yanru Zhang, Hong Chen

**Affiliations:** 1 Institute of Landscape Ecology of Montane Horticulture, Southwest University, Chongqing, 400716, China; 2 Department of Botany, College of Horticulture and Landscape Architecture, Southwest University, Chongqing, 400716, China; Landcare Research, New Zealand

## Abstract

**1:**

This study investigated 15 coexisting dominant species in a humid subtropical evergreen broad-leaved forest in southwest China, consisting of long-lived pioneers and climax species occurring in natural and disturbed regimes. The authors hypothesized that there would be non-tradeoff scaling relationships between sprouting and seed size among species, with the aim of uncovering the ecological relationship between plant sprouting and seed characteristics in the two functional groups.

**2:**

The sprouting variations of the species were initially examined using pairwise comparisons between natural and disturbed habitats within and across species and were noted to show a continuum in persistence niches across the forest dominants, which may underlie the maintenance of plant diversity. Second, a significantly positive, rather than tradeoff, relationship between sprout number and seed size across species within each of the two functional groups was observed, and an obvious elevational shift with a common slope among the two groups in their natural habitat was examined. The results indicate the following: 1) the relationship of seed size vs. sprouts in the natural habitat is more likely to be bet-hedging among species within a guild in a forest; 2) climax species tend to choose seeding rather than sprouting regeneration, and vice versa for the long-lived pioneers; and 3) the negative correlation between sprouting and seed dispersal under disturbed conditions may imply a tradeoff between dispersal and persistence *in situ* during the process of plant regeneration.

**3:**

These findings may be of potential significance for urban greening using native species.

## Introduction

Plant regeneration is an important ecological process for a forest ecosystem in which the sprouting and seeding of woody species is involved [1]. As a mechanism for shaping community dynamics [2], plant regeneration is important for the succession of forest communities and for the stability and restoration of vegetation in a forest ecosystem following various disturbances. As new architectural units, species sprouts [3] have been widely perceived as key functional traits [4]–[6] linked to plant life-history strategies [7], [8], functional types [9], [10] or population persistence [11], [12]. Using sprouts, some damaged trees can occupy their original space niche rapidly and eventually reach the forest canopy again [13].

Furthermore, sprouts, as one of the two plant regenerative traits [4], have been reported to correlate in part to seed size. For example, sprouters would most likely be expressed in seed fecundity [14]. Moreover, it has been argued that there is a tradeoff between sprouts and seed size [15], [16] due to competition for resources between vegetative and reproductive growth [14] relative to starch-tissue content [17]. Plants that sprout vigorously tend to be poorer seed recruiters than non-sprouters [8], and non-sprouters produce more seeds than congeneric sprouters growing at the same sites [8], [17]. Nevertheless, others have found no correlation between the two variables (e.g., [18]). In brief, few significant associations have been reported thus far.

In contrast, because both seeding and resprouting are all ascribed to plant regeneration [4], we may infer that seed size correlates with sprouting [19], [20]. In addition, in fluctuating environments, seeding shows bet-hedging [21], [22], which remains correlated with seed size among community species [23]–[25] during the process of seed germination as an evolutionarily stable strategy to control the plant population (or ESS, e.g., [26]). With respect to resprouting, Nzunda *et al*. [16] recently suggested a bet-hedging model between occasional events and fixed interference in the storage reserve in good resprouting species. Based on these reports, we may hypothesize that plant sprouting may show a pattern of bet hedging in response alternating seeding, which eventually results in a positive scaling relationship, instead of a tradeoff, between plant sprout and seed size.

Moreover, interspecific and intraspecific differences in sprouting ability have been observed [2], [7] that were due to various ecological factors [27]–[30] or the biological properties of the plants themselves [31]–[33]. For example, as an example of the latter, light-demanding species generally show enhanced resprouting abilities compared with shade-tolerant species [18], and species tend to be more widely distributed in less-productive sites [17]. It is reasonable to infer that the connections between sprouting and seed traits may be intrinsically variable in different functional plants (i.e., guilds). An examination of these properties may be vital to understand the regenerative ecology of particular species.

However, direct evidence on the relationship between sprouts and seed size in woody species remains insufficient. Furthermore, much of the existing research on regeneration following disturbances has focused on the role of pioneer species (e.g., [2], [34]–[36]). There have been few comparative studies among long-lived pioneers (LP) and climax species (CS; however, see [37]), which may be of practical value for forestry (see the summary in [38]).

The current study employed a subtropical evergreen broad-leaved forest in Mt. Jinyun Nature Reserve in China. We monitored the sprouting performance of 15 dominant species with varying seed size living in both natural and disturbed areas, and we analyzed intraspecific variations in sprouting responses to disturbances for the 15 species by examining the relationship between species individual sprout number and seed size among the two groups. Finally, we examined the associations among life-history attributes of the species, such as plant height, starch amount in current-year shoots, individual seed size, and appendages per seed carried (a parameter characterizing seed-dispersal ability that may be correlated in part with sprouting, see [39]). The primary objectives of this study were the following: 1) compare the sprouting performance within species across disturbance gradients; 2) test the expected ecological relationships between species' sprouting behaviors and seed traits among CS and LP.

## Materials and Methods

### Study site and vegetation

The study area is located in the Jinyunshan National Nature Reserve (29°50′N, 106°24′E) in southwestern China, covering about 14 km^2^ in total. The elevation within the reserve ranges from 180 to 951.5 m. The climate is of a typical subtropical monsoon type, characterized by mid-subtropical monsoons with a rainy, hot summer and a dry, warm winter. The mean annual rainfall is 1143.1 mm, which primarily falls in summer; thus leading to a relatively high mean annual relative humidity of *c*. 85%. The annual mean temperature is 17°C, with annual effective accumulated temperature (>10°C) of *c*. 6000°C, a maximum annual frost-free period of 334 d, and a mean annual sunshine time of only 1160 h. The most common soil type is acid yellow (mean pH value is 4.36) developed from Triassic quartz sandstone, carbonaceous shale and argillaceous sandstone, which contains a total content of 0.0985% nitrogen, 0.040% phosphorus, 1.413% kalium and 2.099% organic matter [40],[41]. In addition, Mt. Jinyun is both a nature reserve and a famous scenic area where there are many signs of human habitation, such as roads, farmhouses, and hotels. The plants near these sites are greatly influenced by human disturbance.

With respect to zonal vegetation, there is a humid subtropical evergreen broad-leaved forest with secondary succession of vegetation in varying stages that primarily result from a forest fire (∼300 years ago), mining (∼50 years ago), and an abandoned tea garden (∼20 years ago). The forest shows an obvious vertical structure, primarily comprising Fagaceae, Lauraceae, Theaceae, Symplocaceae, and Elaeocarpaceae [42]. The first tree layer is generally 15–20 m high with a few trees taller than 25 m. This tree layer is chiefly dominated by Fagaceae and Theaceae species that average 15 m in height and range from 25–35 cm in diameter at breast height, interspersed with large Elaeocarpaceae, Lauraceae and Symplocaceae trees that also influence the forest community. The second shrub layer is typically dominated by large shrubs or saplings that are often less than 3 m in height [40] and include species that are common in the sunny bare soil or shaded understory.

### Materials and study design

Fifteen dominant or co-dominant species in the tree layer and shrub layer were selected as target species that represented, to some extent, the characteristics of the evergreen broad-leaved forest. The 15 species included three Symplocaceae species, four Theaceae species, three Elaeocarpaceae species, two Lauraceae species, two Fagaceae species, and one Rubiaceae species. They are well-layered in the forest community, and *Castanopsis fargesii*, *Castanopsis carlesii*, *Elaeocarpus duclouxii*, *Machilus nanmu*, *Symplocos laurina* and *Gordonia acuminata* belong to the first tree layer, *Symplocos setchuanensis*, *Adinandra bockiana*, *Neolitsea aurata*, *Adinandra bockiana* and *Sloanea leptocarpa* belong to the second, and *Eurya loguiana*, *Camellia tsofuii*, *Aidia cochinchinensis* and *Symplocos lancifolia* belong to the third [41], [43]. We designated these selected species as either climax species or long-lived pioneers based on their role in the forest community; the dividing approach for climax species and long-lived pioneers followed criteria used in the literature [43], [44] and personal observations.

The field investigations were performed between April 2011 and May 2011 using stratified random sampling to arrange five sample plots (covering about 15 ha in total that were separated from each other by 400–600 m) in a sampling area of about 100 ha. The habitats of individual plants were divided into two types: disturbed habitat (DH; defined as a forest edge within 10 m of buildings, squares, or main roads), which was more strongly affected by human activities, and natural habitat (NH; greater than 50 m from the sources of interference), which was little influenced by human. The plots in the two types of habitats were kept as uniform as possible with respect to altitude, slope position and aspect based on target plants and plant habitat category.

This paper used sprout number to measure sprouting capability, which is the most commonly adopted method (c.f. [45], [46]). Twenty to forty trees per plot were selected randomly to record sprout number per individual in the field. For the purposes of the census, we recorded “distinguished sprout” by specifically referring to the living shoots originating from the trunk [46], and the main stems branching from the trunk were not included.

### Ethics statement

The field studies for each site were permitted by the staff of the Jinyunshan National Nature Reserve, and this study did not involve endangered or protected species.

### Data analyses

In view of the variation in sprout data of interspecifics and intraspecifics under varying habitats, in prior to analyses of the measured traits we conducted nested ANOVAs to test variability. The results showed that both species and habitat influences sprout number markedly (F = 45.630, p<0.001 and F = 6.078, p = 0.014, respectively). Moreover, as a variable, species contributed the largest variance (87.7%) of sprouting data, following by habitat (11.5%) and the individual plant of the same species under the same habitat contributed the least to variation (0.8%). These results showed that sprout data may be compared among species, regardless of their intraspecific variations in different habitats. Data were meaned across individuals of the same species living in the same habitat and log_10_-transformed to improve normality prior to analyzing for the scaling relationships.

The scaling relationships between sprout number and seed size (i.e., seed mass, referring to individual seed dry mass, as in [47]) were analyzed using a Model Type II regression method, with allometric slopes of particular interest calculated as Standardized Major Axes (SMA; [48]). The heterogeneity of regression slopes and the common slopes were tested following the methods of [49], and the shifts between lines fitted to groups sharing a common slope (y-intercept) were examined using ANOVAs. The above allometric parameters were conducted using (S)MATR [48], [50].

Although sprouting capability showed little phylogenetic conservatism [5], we nevertheless performed phylogenetic independent contrasts (PICs) analyses for the species used in the current study to determine whether a correlation between seed size and sprouts was biased by phylogenetic signal. The phylogenetic trees were constructed following Phylomatic (version 3; [51]), and PICs were conducted using the phylogenetic comparative methods of COMPARE (4.6b; [52]) for the LP and CS species.

In addition, to assess differences within species in the two habitat conditions, t-tests were conducted using Statistica 6.0 (Statsoft I 2001: Tulsa, Oklahoma). To detect broad correlations in variation among sprouting traits and plant seed traits, we performed a principal component analysis (PCA). Six statistics were employed to perform the PCA for 15 species. With the exception of two sprouting variables, the remaining four parameters were seed size (a life-history trait related to successional trade-offs), plant height (a parameter that has been shown to be strongly associated to the coexistence of pioneer and shade-tolerant tree species by [37]; sprouters are typically short plants, such as shrubs or bushes, whereas non-sprouters are commonly tall trees [11], [53]), seed appendage per seed carried (taken from [39]), and starch content in shoot pith (estimated roughly using a scale of 1–5, denoting the least to the most amount based on anatomical sections of current-year shoots observed under a microscope). DCA (detrended correspondence analysis) was performed prior to PCA to determine the ordination model (i.e., unimodal or linear) to be adopted following the requirement of [54], using Canoco for Windows 4.5 software. Because all lengths of the gradient were less than 3 (the largest being 0.863), a linear multivariate PCA approach was chosen to examine the associations among the multiple variables including sprout number in NH and DH, seed size, seed appendage per seed carried and starch content in shoot pith and plant height. Of these variables, the first two were assigned as environmental variables and the remaining as species variables for the PCA. For the PCA, the species data were log-transformed, and the focus of scaling was on inter-species correlations. The species scores were divided by the standard deviations, and sample data were centered and standardized, whereas species data were centered by species. The PCA ordination biplot was created using CanoDraw 4.0.

## Results

### Sprouting characteristics under disturbed and natural habitats

The mean numbers of sprouts per trunk for the 15 species in DH and in NH are summarized in Table 1. Five of the species (*Gordonia acuminate*, *Machilus nanmu*, *Symplocos laurina*, *Camellia tsofuii*, *Elaeocarpus duclouxii*) showed more sprouts in NH than in DH; in particular, the former three were significantly different (t-test, p<0.001) and showed sprouting numbers that were 1.8-, 1.9-, and 5.9-fold greater than in the DH. The remaining 10 species showed fewer sprouts in NH than in DH. The sprout numbers for different tree species within the same habitat were also different. The biggest sprout number in the DH was that of *Symplocos lancifolia* (8.64), followed by *Adinandra bockiana* (8.57); the lowest two were *Symplocos laurina* and *Aidia cochinchinensis*, with only 1.6 and 2.56, respectively.

**Table 1:** The species properties and the mean sprout number per individual (mean±SE) in natural habitat and in disturbed habitat for the 15 species studied.

Species	Family	Height	Group	Abbr.	NH	DH	DH/NH
*Castanopsis carlesii*	Fagaceae	Mt	CS	Cc	3.37±0.39 ***	7.05±2.06	2.09
*Eurya loguiana*	Theaceae	Ls	LP	El	3.61±0.75 ***	6.11±1.89	1.70
*Neolitsea aurata*	Lauraceae	St	CS	Na	3.24±0.41 ***	5.44±1.08	1.68
*Elaeocarpus japonicus*	Elaeocarpaceae	Mt	CS	Ej	3.00±0.46 ***	5.00±0.92	1.66
*Aidia cochinchinensis*	Rubiaceae	Ls	CS	Ac	1.67±0.29 *	2.56±0.67	1.52
*Sloanea leptocarpa*	Elaeocarpaceae	Mt	CS	Slc	2.80±0.75 ***	4.02±3.00	1.43
*Castanopsis fargesii*	Fagaceae	Lt	CS	Cf	2.80±0.42 ***	3.82±0.71	1.36
*Adinandra bockiana*	Theaceae	St	LP	Ab	6.35±1.54 ***	8.57±2.72	1.35
*Symplocos lancifolia*	Symplocaceae	St	LP	Slf	6.56±2.12 **	8.64±3.33	1.32
*Symplocos setchuanensis*	Symplocaceae	Mt	CS	Ss	3.23±1.51 ns	4.07±1.61	1.26
*Elaeocarpus duclouxii*	Elaeocarpaceae	Lt	CS	Ed	5.48±1.14 ns	5.29±2.33	0.96
*Camellia tsofuii*	Theaceae	Ls	CS	Ct	3.46±0.93 ns	3.24±0.36	0.94
*Gordonia acuminata*	Theaceae	Mt	LP	Ga	10.50±2.18 ***	5.83±1.21	0.55
*Machilus nanmu*	Lauraceae	Lt	LP	Mn	9.70±3.77 ***	5.17±1.89	0.53
*Symplocos laurina*	Symplocaceae	Mt	LP	Sla	9.44±2.78 ***	2.16±0.31	0.23

* denotes the p-level of student's t-tests for the species in the two habitats. *  =  p<0.05, **  =  p<0.01, ***  =  p<0.001, ns  =  non-significant. Ss  =  small shrub (≤0.5 m), Ms  =  middle shrub (0.5–2 m), Ls  =  large shrub (2–5 m), St  =  small tree (5–8 m), Mt  =  middle tree (8–25 m), Lt  =  large tree (≥25 m); NH & DH  =  sprout number in natural & in disturbed habitat, respectively; Abbr.  =  the abbreviation of the species studied. CS denotes climax species and LP does long-lived pioneer. The species are sorted according to the column of DH/NH.

With the exception of 3 species (t-test, p>0.05; *Elaeocarpus duclouxii*, *Camellia tsofuii* and *Symplocos setchuanensis*), there were notable differences in paired sprouts for the 12 species between NH and DH (t-test, p<0.05; Table 1). Therefore, human activities affect sprouting capacity of trees in general; however, the effect of this interference differs with species.

### Scaling relationship between sprouting capability and species seed size

**Figure 1:**
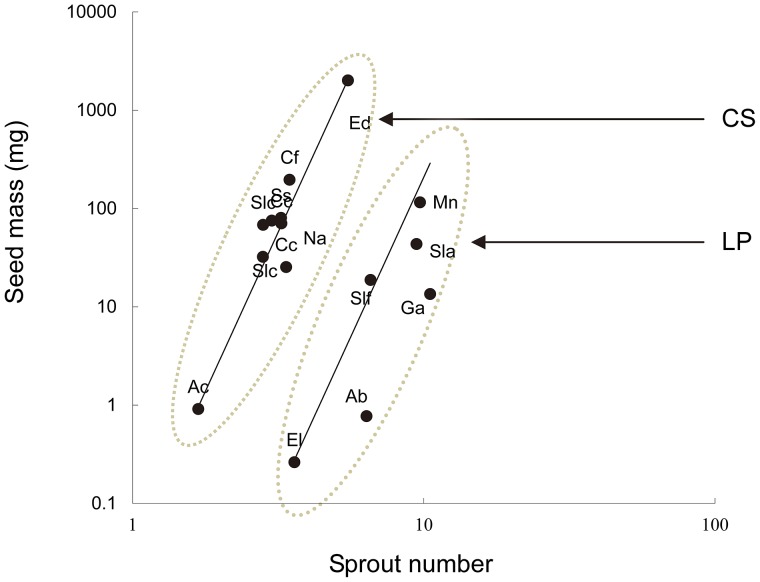
The relationship of seed size vs. sprouts. The bivariate relationship between seed size and trunk sprouting number among climax species (CS) and long-lived pioneers (LP) in natural habitats in a subtropical broad-leaved forest in southwestern China. The two lines in the graph denote the fitted lines to the two functional groups (slope  =  common slope shared by the two functional groups) using the standardised major axis (SMA); broken line, CS species group; solid line, LP group.

Species sprout number in NH did not generally correlate with seed size in pooled data across all 15 species (p>0.05). However, when dividing the 15 species into two groups (CS group and LP group; Table 1), the sprout number closely scaled with seed mass across congeners within a group (r^2^ = 0.714, p = 0.034, slope = 5.884 [2.959 11.702] for the LP group; r^2^ = 0.907, p<0.001, slope = 6.609 [5.049 8.653] for the CS group) with a significant elevation increase (y-intercept*_LP_* = −4.157; y-intercept*_CS_* = −1.479) fitting a common slope ( = 6.476; Figure 1). Second, the sprout number in DH did not correlate with seed mass across species (p>0.05) even when the 15 species were divided into CS and LP (p>0.05). In addition, the above tendencies were maintained in PICs, with the exception of a weak correction in pooled data across the 15 species in DH (Table 2).

**Table 2:** Summary of phylogenetically independent comparative analysis (PICs) for the relationships between sprout number and seed size of the climax species (CS) and long-lived pioneers (LP) studied, and for the pooled data (Pooled) of the two groups.

Habitat	Group	r^2^	p	*a*	*b*
NH	CS	0.807	0.002	6.395	0.057
NH	LP	0.722	0.002	3.326	−0.284
NH	Pooled	0.702	<0.001	4.508	−0.125
DH	Pooled	0.365	0.029	−2.187	−0.670

Letters *a* and *b* are the regressive coefficient and the intercept of the linear regressive equation *Y  =  ax+b*, where *Y* and *x* are seed mass and sprout number, respectively.

These results show that the relationship between sprouting capability and species seed size varies considerably between the two species groups in natural habitats and remained when phylogeny signals were removed. Furthermore, the apparent difference in y-intercept (increase) between the CS and LP groups indicates that the species in the CS group produced larger seeds than those of the LP group for given sprouts.

### The broad trends among sprouting capability, stem and seed traits, and habitats

**Figure 2:**
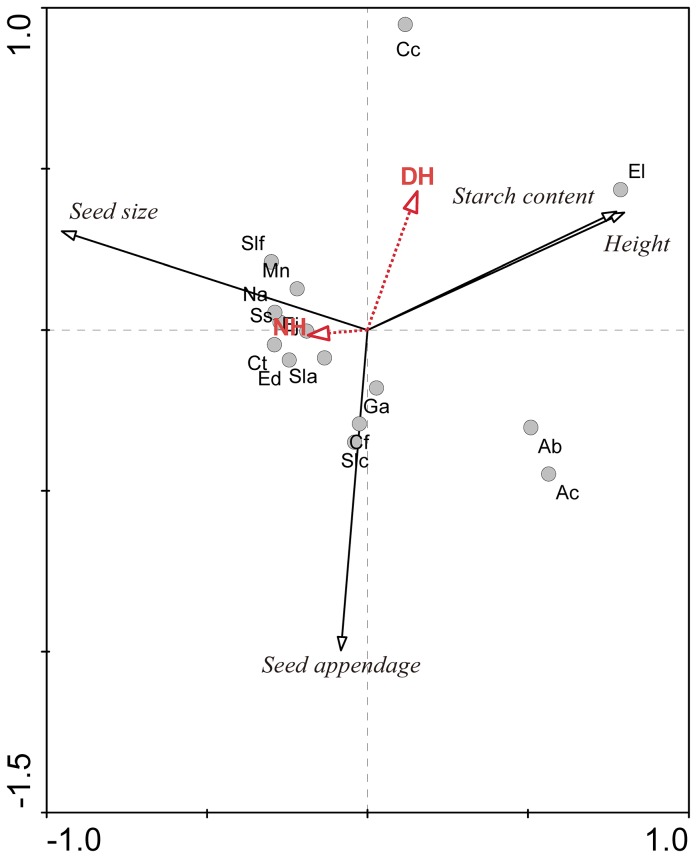
The PCA biplot. Principal component analysis (PCA) based on trunk sprouts in natural sites (NH) and disturbed sites (DH), seed size, seed appendage, starch content in shoot and plant height variables. Circles show sampled species, with abbreviations being as for Table 1.

The PCA graph summarizes the general correlations (Figure 2). The PCA revealed that the first and second PCA axes explained 58.4% and 33.2% of the variability in species data, respectively; 91.6% in total. The variability of NH sprouts paralleled the direction of seed size (Figure 2) but opposed the direction of height and starch content; however, the DH variable was orthogonal to seed size but conversely parallel to the direction of seed appendage. Thus, the NH variable was more closely and positively associated with seed size and correlated negatively with height and starch content. The DH variable correlated strongly with seed appendage per seed carried but was independent of seed size.

## Discussion

We performed intraspecific comparisons of sprouting performance for the dominant species in a subtropical community and tested our predictions on the interspecific relationships between sprouting and seed traits for LP and CS. We observed consistently significant relationships between species sprouting and seed traits; however, the details of these relationships varied based on species and habitat type.

### 1) Contrasting sprouting patterns within and across species between natural and disturbed habitats

The species sprouting patterns differed in conspecifics in different habitat types (consistently showed using t-tests) and also in interspecifics. Some species sprouted vigorously in natural habitats; however, others showed enhanced sprouting in disturbed habitats. The intraspecific sprouting pattern shows that, among the species measured, the majority of species were sensitive to disturbance, indicating that disturbance affects sprouting in plants.

Moreover, the differentiation among dominant species in a forest may be of particular significance due to their leader functions in a forest when considering spatial coexistence among species [55] and the dynamics of the community structure [56]. Some species sprouted more in DH whereas others performed better in NH, as mentioned above, we believe this diverse sprouting property in dominant species forms a vegetative regenerative spectrum, which lays the foundation for them to cope with various natural- or human-derived disturbances, and presumably, it also reflects a bet-hedging sprouting strategy at the species level within a community (c.f. [57]). Thus, the marked differences in sprouting performance may be critical for the dominant species to colonize gaps [58] in a complementary way and may enable maintenance of tree diversity following disturbances, based on the “alternate trait axis” argument [2].

Nevertheless, it is difficult to explain the origin of the difference in sprouting behaviors of some species that sprouted less in disturbed habitats, as observed here in this study. Further research such as investigation for underground clone structure is suggested.

### 2) Relationship between seed size and sprout number

Larger-sized species tend to produce more sprouts among species within a functional group in an undisturbed habitat; however, this relationship did not reach significance when all species were pooled, indicating that natural guild species may evolve the potential to reproduce vegetatively (bet-hedge rather than reproduce sexually) in response to environmental cues [57]. We believe that this result is reasonable based on two lines of reasoning. First, the observed sprouting continuum across dominant species along the disturbed gradient provided a necessity for species hedging their evolutionary bets in varying environments [59]. Second, woody plants are perennial and not annual. The sprouts on trunks were the sprouts saved for years that eventually become the biological “bet” and enable a positive relationship between seed size and sprouts; hence, there is no need for to trade-off their limited resources accumulated within one year. Thus, this strategy may be viewed as the integration of bet-hedging between sprouting and seeding during long-term regenerative evolution.

Furthermore, based on the equal sprouting number on trunks, climax species tended to show larger individual seeds than long-lived pioneers, revealing a difference between the two species groups with respect of life history strategy under natural conditions. This tendency held true in PIC analyses, revealing its ecological significance. The CS guild may win out in a forest by evolving larger seeds, enabling seedlings to settle and establish in a darker habitat. In contrast to CS, the LP species tended to adopt a vegetative means to enable plant regeneration in brighter sites. They produce more sprouts and smaller seeds under natural conditions; thus, enhanced sprouting ability but weaker seeds.

On one hand, this result is inconsistent with authors who argue that vegetative sprouting should require a tradeoff against sexual reproduction in general and consequently, that poor resprouters should show higher seed mass compared with good resprouters (e.g., [15], [60]). This inconsistency may result from the differences in the type of environment studied (e.g., natural vs. disturbed habitat), the species type (the species focused upon were woody CS & LP) or the variation in seed size due to seed size/number tradeoff (e.g., [39]). On the other hand, the greater mean number of sprouts in LP compared with CS under natural conditions, as observed in our study, indicates a pattern of smaller-seeded species with more sprouts [the LP group in NH showed markedly more sprouts ( = 7.695) than the CS group ( = 3.232; t-test, t = −4.661, p<0.001)], which may also represent a tradeoff to some extent between seed size and sprouts. In addition, this relationship did not hold true for the species in disturbed habitats, as shown both in the PCA graph (Figure 2) and in regression analysis (p>0.05), which is consistent with a previous report [18].

### 3) Associations among NH & DH sprouts, seed dispersal, carbon storage and plant height

PCA showed a clear positive correlation of sprouts in NH with seed size, further verifying the relationship between these two variables (Figure 2). NH sprouting was also negatively correlated with starch content and plant height, showing that good sprouters in natural sites may store starch less and include relatively more short species. The DH sprouts showed contrasting results, supporting some previous reports, such as [60], demonstrating that sprouters show higher starch concentrations in roots.

The DH sprouting variable was inversely correlated with seed appendage (Figure 2), suggesting that good sprouters in DH may be weak seed dispersers and vice versa. This relationship may imply a tradeoff between the space occupied by the plant between far (i.e., seed dispersal) and near (i.e., sprouting). The ecological relationship between seed dispersal and plant sprouting may be an interesting topic that deserves further discussion.

### 4) Ecological and practical implications of the sprouting strategy

The sprouts of dominant species in the natural ecosystem may be very useful when managing forests for products including timber, firewood, edible fruits, and landscape plants, which are some of the different uses of the species observed. Thus, our results are likely to be helpful for silvicultural treatments. For example, the difference in seed size between CS and LP implies different regeneration niches in a subtropical forest and lays a good foundation for a more resilient forest ecosystem apart from human disturbances using complementary mechanisms of responding to a more or less disturbed habitat. Still, long-lived pioneers show some excellent features, such as their fast growth and relative shade tolerance, compared with pioneers, and they have light but strong timber (characteristics proposed as part of the long-lived pioneer syndrome by [38] (pp246), which is superior not to the slow-growing climax species but to the strongly r-selected pioneer. Therefore, LP is an issue of particular concern to foresters when applying “Miyawaki's Method” for reestablishing an urban forest [61].

## Conclusions

We tested and confirmed our hypothesis on the ecological correlation of sprout vs. seed size. Dominant forest species differ in their sprouting capability, build divergent sprouting patterns along disturbed gradients and display contrasting relationships of sprouts vs. seed size among guilds, manifesting in diverse sprouting strategies across woody plants and suggesting a strong selection for species life-histories under varying conditions. These results provide evidence for the evolutionary mechanism and consequences of variations in plant sprouting behavior, which may contribute to the ecology of regeneration in a forest community.
